# A Rare Case of Primary Signet Ring Cell Carcinoma of the Urinary Bladder in a Young Woman

**DOI:** 10.1155/criu/9204085

**Published:** 2025-09-08

**Authors:** Pavlo Synoverskyy, Sameh Hijazi, Thomas Krüer, Axel Haferkamp, Maximilian P. Brandt

**Affiliations:** ^1^Department for Urology, Universitätsmedizin der Johannes-Gutenberg-Universität Mainz, Mainz, Germany; ^2^Department for Urology, Stiftung Mathias-Spital Rheine, Ibbenbüren, Germany; ^3^Pathocom-BAG für Pathologie, Osnabrück, Germany

## Abstract

Primary signet ring cell carcinoma of the urinary bladder (PSRCCB) is an exceedingly rare subtype of urinary bladder carcinoma, comprising 0.12%–0.6% of cases, with a poor prognosis. This case report details a distinctive case of a 32-year-old woman with PSRCCB, presenting without typical risk factors and posing diagnostic and therapeutic challenges. Initial symptoms included urinary tract infection and lower abdominal pain. Imaging and histological assessments identified a mucinous adenocarcinoma with signet ring cell components. The patient underwent curative open partial cystectomy, given her young age and localized tumor, avoiding lymphadenectomy and adjuvant chemotherapy due to complete tumor resection and absence of metastases. Postoperative follow-up showed no pathological findings, underscoring the importance of individualized treatment strategies in rare cancer cases. This case contributes to the limited data on PSRCCB and its management.

## 1. Introduction

Urinary bladder cancer (UBC) is the most common malignancy of the urinary tract [1]. The incidence of UBC increases with age, with over 90% of patients aged 55 and older. Primary adenocarcinoma of the urinary bladder is an uncommon entity, accounting for less than 2% of all UBC [2, 3]. It is rare among younger populations [4, 5]. Primary signet ring cell carcinoma of the urinary bladder (PSRCCB) is very rare, with a maximum of 0.6% of all UBC variants [6, 7]. We want to present a rare case of a young woman with PSRCCB, absence of typical risk factors, and diagnostic and therapeutical challenges during the treatment.

## 2. Case Presentation

**Figure 1:**
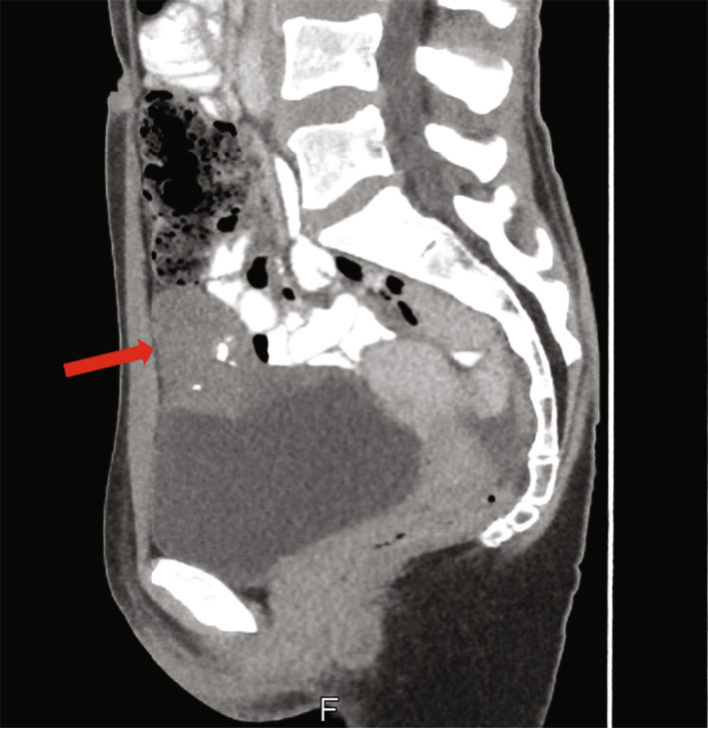
CT of the abdomen in the coronal plane, showing the tumorous mass on the urinary bladder dome with the presence of calcifications (red arrow).

**Figure 2:**
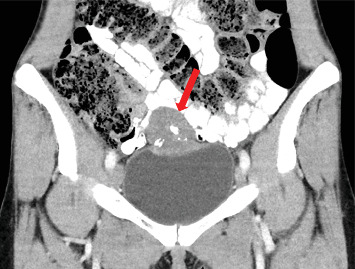
CT of the abdomen in the sagittal plane, showing the tumorous mass on the urinary bladder dome with the presence of calcifications (red arrow).

**Figure 3:**
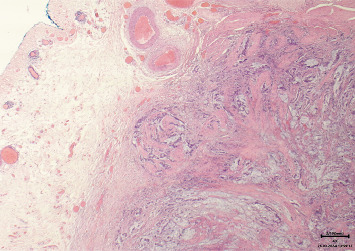
Histological sample of the urinary bladder adenocarcinoma (hematoxylin and eosin stain, 20x magnification).

**Figure 4:**
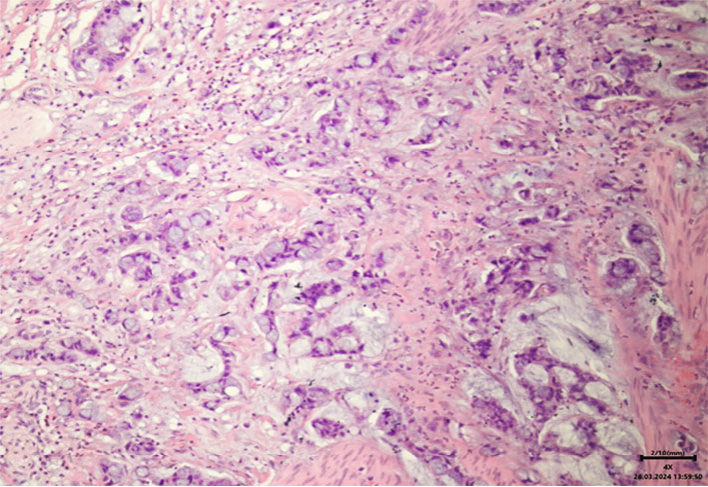
Histological sample of the urinary bladder adenocarcinoma (hematoxylin and eosin stain, 100x magnification).

**Figure 5:**
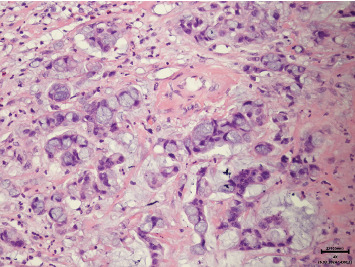
Signet ring cell differentiation (hematoxylin and eosin stain, 200x magnification).

**Figure 6:**
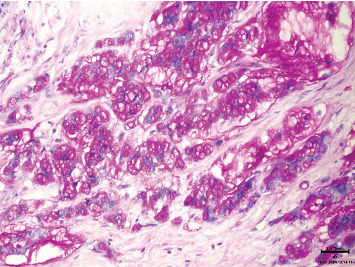
Signet ring cell differentiation (periodic acid–Schiff stain, 200x magnification).

A 32-year-old woman in good general condition was presented to the secondary specialist with symptoms of urinary tract infection and lower abdominal pain, which lasted about a week. No other symptoms were described. The patient's medical history was unremarkable except for endometriosis as a teenager and a surgical removal of a cyst of the right ovary. The patient takes no medications and has no allergies. The family history regarding cancer diseases was unremarkable, and her clinical examination did not reveal any pathological findings. All of her hematological investigations were within normal limits. Urological ultrasound assessment identified an unclear mass on the urinary bladder dome, from which samples were taken after consecutive transurethral resection of the bladder (TURB). The histological assessment showed a mucinous adenocarcinoma with a signet ring cell component. No definitive statement about tumor origin could be made based on the samples taken. For further diagnostics, a computer tomography (CT) scan of the thorax and abdomen was performed, revealing an approximately 5.6 × 4 × 5 cm large smooth-bordered, partially solid, and partially cystic mass with individual calcifications originating from the urinary bladder (Figures 1 and 2). Therefore, a reference to our tertiary specialist unit was performed. Considering the young age of the patient and the absence of lymph node or distant metastases, we opted for a curative open partial cystectomy without neoadjuvant chemotherapy or immunotherapy. Intraoperatively, the tumor was palpable and visible at the dome of the bladder. Neither connection to the urachus nor bowel nor infiltrative growth could be detected. We removed the tumor in toto with a safety margin of minimum of 1 cm. No enlarged lymph nodes were observed in the pelvic area; thus, a lymphadenectomy was not performed. Histologically, PSRCCB with complete resection was confirmed (Figures 3, 4, 5, and 6). Immunohistochemically, the tumor was positive for mismatch repair proteins MSH-2, PMS-2, MSH-6, and MLH-1. A postoperative esophagogastroduodenoscopy (EGD) and coloscopy were performed to exclude an alternative origin of the tumor. No carcinoma was found in both assessments. Conclusively, the fluorodeoxyglucose positron emission tomography (FDG-PET-CT) scan was performed with no sign of locoregionary or distant metastases confirming the primary CT findings. Following an interdisciplinary consensus from our tumor board review and considering limited data availability as well as the complete local resection of the tumor with the absence of metastatic spread, we elected to forego adjuvant chemotherapy as well. The patient was discharged home after a 2-week stay in the hospital in good general condition and was able to return to daily activities 3 days after discharge and take up work after 5 weeks. The follow-up intervals every 3 months included urine analysis and cystoscopy. To the time of publication, two follow-up visits have been performed, both without pathological findings.

## 3. Discussion

Primary adenocarcinoma of the urinary bladder is a rare entity, which requires a different approach to diagnostics and therapy. The location at the urinary bladder dome should be primarily suspected as urachal cancer until further diagnostic clarification is obtained [8]. Differential diagnoses could also include primary adenocarcinoma or metastasis. Especially when encountering a primary urinary bladder adenocarcinoma that is not of urachal origin, it is essential to rule out other potential primary sites, given the extreme rarity of this tumor type as a primary lesion. PSRCCB of the urinary bladder, a rare bladder tumor variant, comprises about 0.12%–0.6% of all bladder cancers [6, 7]. Classified as a subtype of bladder adenocarcinoma, PSRCCB is associated with the poorest prognosis [9]. With a male/female ratio of 3:1 and an average diagnosis age of 60 years [10, 11], our patient combined both untypical age and sex for cancer manifestation of this type.

There are no clinical guidelines and established treatment for PSRCCB to the time of publication. Most of the patients are multimorbid and of advanced age, often with local metastases or tumor in locally advanced stage at the time of diagnosis [10]. As PSRCCB consistently shows resistance to radiotherapy and standard chemotherapy as a primary treatment, a surgical approach with radical cystectomy and lymphadenectomy has proven to have the best outcome and survival rates for such patients [11]. Our decision against a radical cystectomy and for a curative open partial cystectomy was made given the patient's young age, absent comorbidities, and localized tumor aiming at the optimization of the patient's quality of life postoperatively. A decision for partial cystectomy in similar cases was also preferred by other authors [12, 13]. The absence of lymph node involvement macroscopically and in CT findings supported the decision against lymphadenectomy, minimizing surgical morbidity by a young patient.

The decision to forego adjuvant chemotherapy was based on several factors: the complete resection of the tumor, no further tumor findings in EGD and coloscopy, and the absence of metastatic disease in conventional CT and FDG-PET-CT. Moreover, the lack of definitive data supporting the benefit of adjuvant chemotherapy by localized tumor, as well as the rare metastatic spread of PSRCCB, supported our decision [14]. This approach highlights the importance of individualized patient care, particularly when dealing with rare cancer entities where established guidelines do not exist.

## 4. Conclusion

• PSRCCB is a very rare entity, which can be observed in various groups of patients, including young people with unremarkable medical history.
• Quick communication and good cooperation between secondary and tertiary specialist centers (14 days from initial presentation to operation) are crucial for satisfying postoperative outcome.
• An exclusion of an alternative origin of the tumor should be performed. This should include EGD and coloscopy as well as advanced diagnostic imaging.
• Due to the absence of established clinical guidelines for PSRCCB treatment, an individual multidisciplinary approach for operative, radiochemical, and/or immunotherapy, including molecular and cytogenetic studies, should be performed for each patient. Postoperative quality of life and comorbidities as well as the probability of metastatic spread should be considered.

## Data Availability

Data are available on request from the authors.
